# Changes in the Bone Mineral Density after Sleeve Gastrectomy vs. Roux-En-Y Gastric Bypass 2 Years after Surgery

**DOI:** 10.3390/nu14153056

**Published:** 2022-07-26

**Authors:** María-José Castro, José-María Jiménez, María López, María-José Cao, Gilberto González-Ramírez, María de Lourdes Bolaños-Muñoz, Jaime Ruiz-Tovar

**Affiliations:** 1 Research Group “Multidisciplinary Assessment and Intervention in Health Care and Sustainable Lifestyles VIMAS+”, Nursing Faculty, University of Valladolid, 47005 Valladolid, Spain; mariajose.castro@uva.es (M.-J.C.); maria.lopez.vallecillo@uva.es (M.L.); mjcao@enf.uva.es (M.-J.C.); 2 Department of Surgery and Bariatric Surgery, Real San Jose Hospital, Guadalajara 45040, Mexico; gilpchmd@yahoo.com.mx; 3 Department of Neuropsychology and Neurolingüístic, Neuroscience Institute, Universidad de Guadalajara, Guadalajara 44130, Mexico; mariad.bolanosm@academicos.udg.mx; 4 Department of Health Sciences, University Alfonso X, 28691 Madrid, Spain; jruiztovar@gmail.com

**Keywords:** sleeve gastrectomy, Roux-en-Y gastric bypass, bone mineral density, osteoporosis

## Abstract

The aim of this study was to compare the analytical and densitometric changes 2 years after Roux-en-Y gastric bypass (RYGB) and sleeve gastrectomy (SG). A retrospective study of a prospectively collected database was performed. Morbidly obese patients undergoing RYGB or SG, as primary bariatric procedures, were included. Weight loss; analytical levels of parathormone (PTH), vitamin D, and calcium; and densitometric parameters were investigated. In total, 650 patients were included in the study, and 523 patients (80.5%) underwent RYGB and 127 (19.5%) SG. There were no significant differences in excess weight loss at 24 months between both groups. When comparing preoperative and postoperative values, a significantly greater increase in PTH values was observed in the RYGB group, whereas there were no significant differences in calcium and vitamin D levels. The mean *t*-score values decreased after surgery at all the locations and in both groups. The reduction in the *t*-score was significantly greater in the RYGB group at the femoral trochanter and lumbar spine. A decrease in bone mineral density (BMD) was observed after both techniques. The mean BMD decrease was significantly greater in the femoral trochanter and lumbar spine after RYGB.

## 1. Introduction

Bariatric surgery is the most efficient treatment option for patients with severe obesity in whom conservative measures have failed, to obtain significant and maintained weight loss and an improvement of obesity-related comorbidities. Both of the most commonly bariatric procedures, Roux-en-Y gastric bypass (RYGB) and sleeve gastrectomy (SG), are performed with very few complications, low mortality, few readmissions, and a low reoperation rate and show significant short- and mid-term benefits of weight loss and resolution of comorbidities in these patients [1,2,3].

The anatomical changes that are imposed by surgical approaches, which bypass the duodenum and part of the small bowel (i.e., RYGB), lead to a reduction in the amount of nutrients available for absorption. It is likely that nutritional deficiencies (Vitamin B12, iron, calcium, and thiamine) appear after these procedures, and metabolic complications, such as osteoporosis, are a consequence of anatomical and functional changes or of inadequate nutritional supplementation (Vitamin D and calcium) [4,5].

Theoretically, patients undergoing a RYGB tend to have a greater decrease in nutrients and trace elements than procedures involving just a reduction in the gastric volume (i.e., SG). The main differences in the postoperative micronutrient deficiencies appear in lipophilic vitamins (A, D, E, and K) because of the selective fat malabsorption induced by the RYGB; these nutrients main absorption is produced in the duodenum (calcium and iron), which is bypassed in the RYGB. Consequently, levels of parathyroid hormone (PTH) usually increase to maintain the serum calcium levels, at the expense of calcium resorption from the bone, which may lead to osteopenia or osteoporosis status [6].

The aim of this study was to evaluate and analyze the impact of the two most common surgical approaches performed at our institution (SG and RYGB) on analytical parameters of bone metabolism (calcium, vitamin D, and PTH), and bone mineral density, as assessed by densitometry.

## 2. Materials and Methods

A retrospective study of a prospectively collected database was performed. Morbidly obese patients undergoing RYGB or SG, as primary bariatric procedures, were included.

### 2.1. Preoperative Evaluation

Preoperative assessment included an abdominal ultrasound; an upper gastrointestinal endoscopy with a Helicobacter pylori diagnostic test; functional respiratory tests; and a nutritional analytical evaluation that included serum levels of calcium, iron, vitamins A, D, E, and B12, and folic acid. Psychiatrists assessed interviews to evaluate the implication of the patient in the postoperative course. Patients received information about possible perioperative complications, and necessary postoperative nutritional supplementation.

### 2.2. Selection Criteria of Surgical Technique

All patients considered for bariatric surgery had either body mass index (BMI) ≥ 40 kg/m^2^ or a BMI ≥ 35 kg/m^2^ with inadequately controlled obesity-related comorbidities (e.g., T2DM, HT, DL, or SAHS). Patients with a BMI over 50 kg/m^2^, high surgical risk due to comorbidities, a known severe nutritional deficit, comorbidities requiring chronic medication, inflammatory bowel disease, the presence of gastroduodenal pathology requiring endoscopic follow-up, and a probability of technical difficulty prior to surgery (multiple previous surgeries or known anatomic modifications) were assigned for the SG procedure. Patients with gastroesophageal reflux disease (GERD) were excluded from SG. In the rest of the patients, RYGB was considered the bariatric technique of choice.

### 2.3. Surgical Techniques

All procedures were performed laparoscopically. Five ports were used both in SG and RYGB; two 12-mm ports were located in the right and left hypochondria, two 11-mm ports were located in the epigastrium and subxiphoideal region, and one 5-mm port was located in the left flank. In SG, a longitudinal resection from the angle of His to approximately 3 to 5 cm orally to the pylorus was performed using a 36-Fr bougie inserted along the lesser curvature. A staple line reinforcement was performed with a continuous oversewing absorbable barbed suture (V-loc 3/0, Covidien, Minneapolis, MN, USA) before extracting the bougie. 

RYGB was performed with a 6-cm-long gastric pouch, calibrated with a 36-Fr bougie. Gastrojejunal anastomosis was also calibrated between 2 and 3 cm. The length of the alimentary limb was 150 cm, and the length of the biliopancreatic limb was 60 cm. The jejuno-jejunal anastomosis was calibrated at 6 cm. 

Before hospital discharge, both groups of patients received identical postoperative counseling, and multivitamin supplementation (Multicentrum, GSK, Philadelphia, PA, USA, 2 tablets/day) and diet. This multivitamin supplementation included calcium and vitamin D. Patients were encouraged to play sports or to do outdoor activities in all seasons.

### 2.4. Follow-Up

All the patients were followed up by the surgeon and endocrinologist one, 6, 12, 18, and 24 months after surgery, and later on yearly. During the follow-up, anthropometric parameters and comorbidities resolution were evaluated.

Medical treatment, such as with antidiabetic, antihypertensive, and hypolipemiant drugs, was adjusted according to the current needs of the patient. The nutritional status of the patients was evaluated by the endocrinologist with analytical blood tests. Deficiencies were supplemented according to the results obtained. The need for treatment with a CPAP mask was evaluated by the pneumologist, according to the results of polysomnography. 

### 2.5. Variables

All the variables analyzed for this study were determined at baseline (preoperative values) and 24 months after surgery. Anthropometric variables included weight, BMI, and excess weight loss. Analytical levels of parathormone (PTH), vitamin D, and calcium were investigated. The number of patients with calcium and vitamin D deficiencies and requiring specific supplementation were calculated. Densitometric parameters included BMD, assessed as the *t*-score at the femoral neck, the femoral trochanter, and the lumbar spine.

### 2.6. Bone Densitometry

Bose densitometry was performed using a LUNAR iDXA (GE Healthcare, Chicago, IL, USA). The densitometer performed a dual energy X-ray absorptiometry. The programs OneScan™ (LSPediA, West Bloomfield Township, MI, USA), Composer™ (Hartford, WI, USA), and Connectivity™ (Boston, MA, USA), belonging to the Prodigy™ (Boston, MA, USA) system, were used for the computer processing. The lumbar spine (L1–L4), femoral neck, and femoral trochanter BMD were measured. The t score was defined as the number of standard deviations above or below the mean for a healthy 30-year-old adult of the same sex and ethnicity as the patient. 

### 2.7. Biochemical Parameters

25-OH-D3 (vitamin D) was measured with isotope-dilution liquid chromatography–tandem mass spectrometry (Sigma Aldrich™, St. Louis, MI, USA). Intact PTH was determined by Elecsys 2010 analyzer (Roche Diagnostics GmbH, Hong Kong, China).

### 2.8. Statistical Analysis 

Data analysis was performed using IBM SPSS v. 22.0 software (IBM, Armonk, NY, USA). The results were expressed as means ± SD or number and percentages. Student *t* tests were used to compare quantitative variables between groups. A *p* value < 0.05 was considered to be statistically significant. 

## 3. Results

### 3.1. Patients Characteristics

A total of 650 patients were included in the study. 523 patients (80.5%) underwent RYGB and 127 (19.5%) underwent SG. There were no significant differences in age and gender between the groups. However, the groups were significantly different in weight and BMI. There were no significant differences in the distribution of preoperative comorbidities between groups (Table 1). 

### 3.2. Preoperative Analytical and Densitometric Values

There were no significant differences in baseline calcium, vitamin D, and PTH values between groups. Similarly, significant differences in the *t*-score, determined at the femoral neck, the femoral trochanter, and the lumbar spine, were not observed (Table 2).

### 3.3. Changes in Anthropometric Parameters 2 Years after Surgery

The RYGB group showed a significantly lower mean BMI (28.1 +/− 4.3 Kg/m^2^ in RYGB vs. 30.2 +/− 5 Kg/m^2^ in SG group (*p* = 0.01). However, there were no significant differences in excess weight loss (EWL) between groups (84.7 +/− 21.7 in RYGB and 83.3 +/− 24.4 in SG (*p* = 0.433). Consequently, the differences in BMI 2 years after surgery were mostly based on the preoperative greater BMI among the patients in the SG group. 

### 3.4. Analytical Values of Calcium, Vitamin D, and PTH, and Requirements of Supplementation 2 Years after Surgery

The mean postoperative values of calcium and PTH were within the normal range. However, the mean vitamin D values were in a deficiency range. There were no significant differences in these parameters between groups. Similarly, the percentage of patients requiring vitamin D and calcium specific supplementation, because of previously detected deficiencies, was not significantly different between groups (Table 3).

### 3.5. Bone Mineral Density 2 Years after Surgery

Postoperatively, the *t*-scores determined at the femoral neck, the femoral trochanter, or the lumbar spine show mean negative values but within the normal range. The osteopenia and osteoporosis rate were below 6.5% in all the parameters and in both groups. There were no significant differences in the percentage of patients with osteopenia or osteoporosis between groups (Table 4). 

### 3.6. Changes from Baseline to Postoperative Values in Analytical Parameters and Bone Mineral Density

When comparing preoperative and postoperative values, there was a significant increase in PTH values in the RYGB group. There were no significant differences in calcium and vitamin D levels between groups.

**Figure 1:**
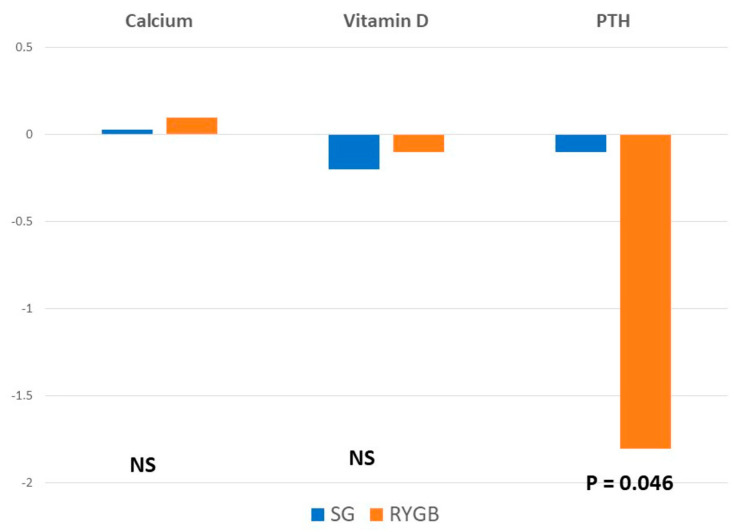
Mean differences between baseline and postoperative values in analytical parameters.

**Figure 2:**
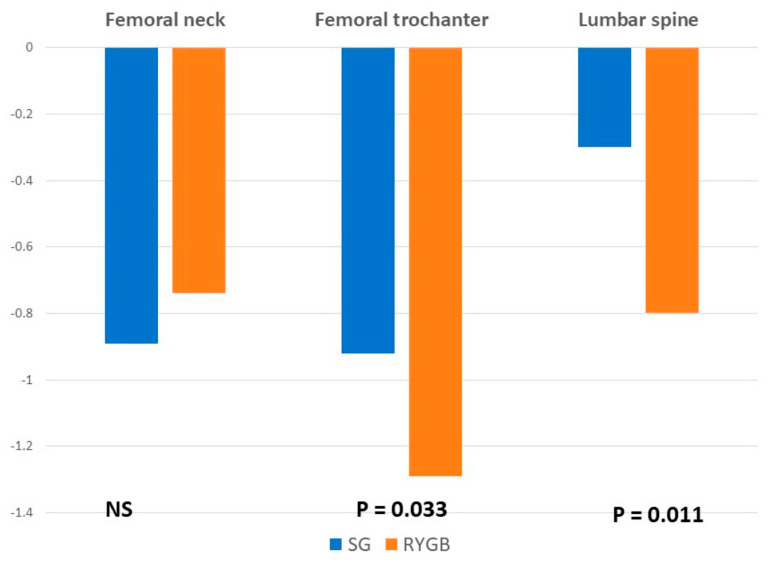
Mean differences between baseline and postoperative values in analytical parameters and bone mineral density.

When analyzing bone mineral density, mean *t*-score values decreased after surgery at all the locations and in both groups. There were no significant differences in the femoral neck values between groups. However, the reduction in *t*-score was significantly greater in the RYGB group at the femoral trochanter and the lumbar spine (Figure 1 and Figure 2).

## 4. Discussion

Several studies have reported a decrease in bone mineral density (BMD) after Roux-en-Y gastric bypass (RYGBP) [7,8,9]. In contrast, the decrease in BMD is more controversial after SG. Some studies describe a decline in BMD [10,11], whereas other authors report an increase in BMD, associated with an augmentation of vitamin D levels, released from the adipose tissue once it decreases due to weight loss. It must be considered that the latter was reported in a Spanish population in the Mediterranean coast, with a high number of hours of sunlight exposure, which probably increased the vitamin D skin absorption [12]. The present study shows a decrease in BMD after both RYGB and SG. 

The clinical relevance of the mean decrease in BMD is that 2 years after surgery, up to 2.1% of patients showed *t*-score values in the range of osteoporosis and up to 5.7% in the range of osteopenia, without significant differences between the surgical procedures. However, the mean BMD reduction was significantly higher among patients undergoing a RYGB at the lumbar spine and the femoral trochanter, without differences at the femoral neck. This is probably a consequence of a higher increase in PTH values in the group of patients with RYGB.

Several reports have described a more pronounced decrease in BMD in the femur than in the spine, revealing that the femur seems to be a more sensible location for BMD changes. This hypothesis has been also confirmed in our study. Fleischer et al. [11] even reported a decrease of 9.2% in the femoral neck but did not observe any changes in the lumbar spine. A potential explanation for why densitometers detect different BMD in the lumbar spine and in the proximal femur is that they are unable to accurately measure changes in adipose tissue density and distribution following bariatric surgery [13].

Most studies have reported an association between weight loss and decrease in BMD, showing a more pronounced BMD reduction in those patients with greater weight loss [8]. However, some authors have described that this association is valid with proximal femur measurements but not for those values obtained from the lumbar spine. We failed to confirm this affirmation as we could not demonstrate any association with the measurements performed in the lumbar spine, the femoral neck, or the femoral trochanter [14].

To adequately evaluate bone metabolism after surgery, it is essential to analyze the serum vitamin D, PTH, calcium, and phosphate levels. These parameters are crucial for BMD maintenance in periods of weight loss and catabolic state. Some morbidly obese patients present preoperative nutrient deficiencies, secondary to their alimentary disorders. In our series, mean baseline values of vitamin D were below the normal range. The cause of this deficiency can be partly attributed to vitamin D abduction in the adipose tissue, but inadequate nutrition and low sunlight exposure due to sedentarism and psychological lability, leading these patients to a tendency of living indoors, also contribute to the vitamin D carency [15]. However, the prevalence of postoperative abnormalities is usually higher [15,16], mostly associated with a decrease in food intake and with the anatomical changes in the gastrointestinal tract, presenting procedures that bypass the duodenum and part of the small bowel, the highest risk [16,17]. Therefore, given the reduction in calcium absorption, the organism starts bone resorption to maintain serum calcium within the normal range. 

This bone resorption is mediated by PTH; thus, PTH levels increase in the postoperative course [7]. In our results, we could not observe significant modifications in calcium and vitamin D values in any of the groups. In the SG group, PTH did not display relevant alterations either, but the increase in PTH was significantly higher in the RYGB group, which is probably due to a greater decrease in BMD in these patients. Given that the postoperative mean vitamin D values are still below the normal range in both groups despite the prescribed supplementation, it is probably advisable to prescribe vitamin D supplements to all the patients routinely and even at higher doses in patients undergoing RYGB.

Vitamin D levels have been correlated with the number of hours spent outside [18]. Our patients were stimulated to perform outdoor activities, but the real activity they perform still remains unknown for the medical staff. As they showed sedentarism habits preoperatively, it is difficult to believe that they show intense physical activity after surgery. Thus, the periodical monitoring of physical activity using pedometers would be advisable to identify subjects with a reduction in sunlight absorption of vitamin D and at risk of developing postoperative secondary hypoparathyroidism and a possible decrease in BMD.

In this way, another factor involved in bone loss after surgery is a lower mechanical load [19]. It seems to be especially important in the variation of bone mass. Some authors found that reductions in bone mineral density were associated with weight loss and not with variations in vitamin D [20]. These correlations suggest that the large reductions in weight caused by bariatric surgery imply a lower mechanical load, which may be a relevant factor for bone mineral content reduction.

Although little information is currently available about this topic, some studies show that fracture probability within 10 years increases 2.3 times after bariatric surgery. A pathologic fracture in patients with osteopenia or osteoporosis is the real clinical relevance of determining BMD. Bioelectrical impedance has appeared as a reliable tool to detect osteopenia and osteoporosis; subjects with a normal bone density showed 3.2 kg of bone mass (measured by bioelectrical impedance), while subjects with 2.7 kg and 2.6 kg suffered osteopenia and osteoporosis, respectively [21]. A previous study of our group determined that patients undergoing a one-anastomosis gastric bypass decreased bone mass up to 2.73 kg and 2.67 kg at 6 and 12 months of surgery, reporting that patients are likely to suffer from osteoporosis. In fact, at 6 months after surgery, 60.3% of the patients showed values ≤ 2.7 kg bone mass, increasing to 64.5% 12 months after surgery. These data do not significantly differ from the reported results after RYGB or SG [22]. At our institution, the first BMD determination by densitometry is performed 2 years after surgery and is repeated every 2 years. Given that the decrease in BMD is relevant 6 months after surgery, the time to conduct the first postoperative examination should be probably brought forward or other diagnostic tools should be used, such as bioelectrical impedance, as a screening for early osteopenia or osteoporosis detection.

Health professionals should use strategies to prevent these disadvantages, and these should be applied mainly during the first 6 months of the surgery since it is at this point that major reductions in bone mass occur. A treatment that seems to be especially effective is to perform exercise after surgery. Performing exercise reduces the loss of bone mineral density [23,24]. In addition, the type of exercise that is performed is determinant since it seems that resistance training takes on great importance for preventing losses of bone mass [25].

### Limitations

In our study, the number of patients was not comparable between groups as the number of patients undergoing RYGB was significantly greater. In addition, RYGB was the bariatric procedure of choice at our institution, and the election of performing a SG was based on high surgical risk, super-obese patients, or patients requiring a chronic intake of medication, whose absorption could be significantly altered with the RYGB. Consequently, the SG group is basically a collection of “outsiders”. This is not only represented by the baseline differences between groups but by the high comorbidities, previous abdominal surgeries with eventual anatomic modifications of the gastrointestinal tract, or the chronic intake of medication, which may alter or interfere with micronutrient absorption. Thus, the results we obtained in the SG group might not be extrapolated to the general population. Future studies, including a similar number of patients in each group and with similar indications for the bariatric approached, must be conducted to confirm the present results.

## 5. Conclusions

A decrease in bone mineral density (BMD) was observed after both techniques. The mean BMD decrease was significantly greater in the femoral trochanter and the lumbar spine after RYGB. There were no significant differences in the percentage of patients with osteoporosis or osteopenia between surgical approaches. 

## Figures and Tables

**Table 1:** Baseline distribution of age, gender, comorbidities, and weight between groups.

	RYGB *n* = 523	SG *n* = 127	*p*-Value
Age	45.3 ± 10.15	45.13 ± 10.82	NS
Weight	120.14 ± 20.6	132.93 ± 26.69	0.001
BMI	44.55 ± 8.1	47.22 ± 7.61	0.001
Male/Female	31%/69%	29%/71%	NS
Type 2 diabetes mellitus	24.3%	29.14%	NS
Hypertension	31.9%	29.14%	NS
Dyslipidemia	28.9%	25.2%	NS

**Table 2:** Baseline distribution of analytical and densitometric parameters between groups.

	RYGB *n* = 523	SG *n* = 127	*p*-Value
Calcium	9.300 ± 0.7	9.3 ± 0.4	NS
Vitamin D	25.8 ± 10.5	29.6 ± 11.6	NS
PTH	60.5 ± 25.9	65.9 ± 39.2	NS
*t*-score Femoral Neck	0.004 ± 1.03	0.31 ± 1.8	NS
*t*-score Femoral Trochanter	0.32 ± 0.97	0.12 ± 1.5	NS
*t*-score Lumbar Spine	−0.15 ± 1.26	−0.29 ± 1.3	NS

**Table 3:** Analytical values and percentage of patients requiring specific supplementations 2 years postoperatively.

	RYGB *n* = 523	SG *n* = 127	*p*-Value
Calcium supplementation	11.5%	8.7%	NS
Vitamin D supplementation	41.9%	33.9%	NS
Calcium (mg/dL)	9.2 ± 0.4	9.3 ± 0.4	NS
Vitamin D (U/L)	25.9 ± 11.3	29.8 ± 15.3	NS
PTH (U/L)	62.3 ± 28	66 ± 59.7	NS

**Table 4:** Two years postoperative densitometric values and osteoporosis and osteopenia rates between groups.

	RYGB *n* = 523	SG *n* = 127	*p*-Value
*t*-score Femoral Neck	−0.7 ± 1.04	−0.59 ± 1.4	NS
*t*-score Femoral Trochanter	−0.87 ± 1.24	−0.8 ± 1.3	NS
*t*-score Lumbar Spine	−0.95 ± 1.38	−0.59 ± 1.5	NS
*t*-score Femoral Neck -Osteoporosis (<−2.5) -Osteopenia (−1−2.5)	4 (0.76%) 27 (5.2%)	1 (0.78%) 6 (4.7%)	NS NS
*t*-score Femoral Trochanter -Osteoporosis (<−2.5) -Osteopenia (−1−2.5)	4 (0.76%) 30 (5.7%)	1 (0.78%) 6 (4.7%)	NS NS
*t*-score Lumbar Spine -Osteoporosis (<−2.5) -Osteopenia (−1−2.5)	11 (2.1%) 26 (4.9%)	2 (1.6%) 7 (5.5%)	NS NS
